# 1-Methoxylespeflorin G11 Protects HT22 Cells from Glutamate-Induced Cell Death through Inhibition of ROS Production and Apoptosis

**DOI:** 10.4014/jmb.2011.11032

**Published:** 2020-12-30

**Authors:** Phil Jun Lee, Chau Ha Pham, Nguyen Thi Thanh Thuy, Hye-Jin Park, Sung Hoon Lee, Hee Min Yoo, Namki Cho

**Affiliations:** 1 College of Pharmacy and Research Institute of Pharmaceutical Science and Technology, Ajou University, Suwon 6499, Republic of Korea; 2 Biometrology Group, Korea Research Institute of Standards and Science (KRISS), Daejeon 34113, Republic of Korea; 3 Department of Microbiology and Molecular Biology, Chungnam National University, Daejeon 414, Republic of Korea; 4 College of Pharmacy and Research Institute of Drug Development, Chonnam National University, Gwangju 61186, Republic of Korea; 5 College of Pharmacy, Chung-Ang University, Seoul 06974, Republic of Korea

**Keywords:** 1-Methoxylespeflorin G11, HT22 cells, reactive oxygen species (ROS), apoptosis, mitochondrial membrane potential (MMP)

## Abstract

This study aimed to investigate the neuroprotective effects of 1-methoxylespeflorin G11 (MLG), a pterocarpan, against glutamate-induced neurotoxicity in neuronal HT22 hippocampal cells. The protective effects of MLG were evaluated using MTT assay and microscopic analysis. The extent of apoptosis was studied using flow cytometric analysis performed on the damaged cells probed with annexin V/propidium iodide. Moreover, mitochondrial reactive oxygen species (ROS) were assessed using flow cytometry through MitoSOXTM Red staining. To determine mitochondrial membrane potential, staining with tetramethylrhodamine and JC-1 was performed followed by flow cytometry. The results demonstrated that MLG attenuates glutamate-induced apoptosis in HT22 cells by inhibiting intracellular ROS generation and mitochondrial dysfunction. Additionally, MLG prevented glutamate-induced apoptotic pathway in HT22 cells through upregulation of Bcl-2 and downregulation of cleaved PARP-1, AIF, and phosphorylated MAPK cascades. In addition, MLG treatment induced HO-1 expression in HT22 cells. These results suggested that MLG exhibits neuroprotective effects against glutamate-induced neurotoxicity in neuronal HT22 cells by inhibiting oxidative stress and apoptosis.

## Introduction

Alzheimer’s disease (AD), the most common form of dementia, is a progressive age-related neurodegenerative disease [1, 2]. Oxidative stress and excitotoxicity dysfunction are highly implicated in neuronal loss and synaptic dysfunction, the principal features of AD [3]. Glutamate, a major excitatory neurotransmitter, is responsible for cell survival, migration, and differentiation during brain development. However, an excessive amount of glutamate leads to neuronal cell death through oxidative stress or excitotoxicity [4, 5]. Neurons are particularly prone to produce reactive oxygen species (ROS) and are highly susceptible to redox stress. Hence, AD is known to be associated with the generation of ROS, which leads to the apoptosis of neuronal cells [6, 7]. Furthermore, oxidative stress causes mitochondrial dysfunction; this triggers translocation of the apoptosis-inducing factor (AIF) to the nucleus, resulting in the activation of the caspase-independent apoptotic pathway [5].

The excessive production of ROS promotes phosphorylation of mitogen-activated protein kinases (MAPKs). Thus, suppression of glutamate-induced oxidative stress using antioxidants could be a good strategy for the prevention and treatment of AD.

Flavonoids, naturally occurring polyphenolic compounds, are normal constituents of the human diet and are known for various biological activities [8]. Pterocarpans constitute the second largest group of isoflavonoids. They contain a tetracyclic ring system of benzofuran–benzopyran having two chiral centers in the 6a and 11a positions derived from the flavonoid skeleton [9]. Pterocarpans exhibit numerous bioactivities including antineuroinflammatory, anticancer, antioxidant, antimalarial, and antimicrobial effects [9, 10]. In our study, while screening for a multipotent agent from medicinal plants with both antioxidant and antiapoptotic properties, we found that the methanolic extract of *Lespedeza bicolor* Turcz. (Leguminosae) exerts protective activity in glutamate-injured HT22 hippocampal cells. Pterocarpans from *L. bicolor* are known to possess beneficial effects, particularly anticancer, antioxidant, and neuraminidase inhibitory activities [11, 12]. Previously, we isolated four new pterocarpans from *L. bicolor* that displayed antiproliferative activity in Jurkat cells [13]. However, to the best of our knowledge, the neuroprotective activity of pterocarpans against glutamate-induced toxicity in HT22 hippocampal cells had not yet been investigated. In the present study, we further identified bioactive pterocarpans in *L. bicolor* exhibiting the neuroprotective effect in HT22 cells, a well-established murine hippocampal neuronal cell line for studying neuronal cell death by glutamate-induced oxidative stress [14, 15].

## Materials and Methods

### Isolation of 2-Geranylbicolosin A (1)

The isolation and structure elucidation of compounds **2–8** have been described previously [16]. For the isolation of compound **1**, ethyl acetate fraction (EA) was subjected to silica gel column chromatography (CC) and eluted with CH_2_Cl_2_–MeOH, yielding four fractions (E1–E4). E1 (16.4 g) was further separated using silica gel CC (hexane–ethyl acetate) to yield five subfractions (H1–H6). Seven subfractions (M18–M24) were obtained from subfraction H-1 by RP C18-MPLC. The separation of subfraction M22 (1.5 g) yielded compound **1** (44.2 mg) by semipreparative HPLC using 75% CH_3_CN in H_2_O.

2-Geranylbicolosin A (**1**): brownish-yellow solid; ${\left[\alpha \right]}_{D}^{20}$-163 (c 0.21, MeOH); ECD (Δε +5.85) at 287 nm (MeOH); UV (MeOH) λmax (log ε) 284 (3.98), 230 (4.27) nm; IR (MeOH) vmax 3421, 2969, 2923, 1615, 1462, 1348, 1169, 1128, 1072 cm-1; ^1^H (DMSO-*d*_6_, 400 MHz); ^13^C NMR (DMSO-*d*_6_, 100 MHz) data, see Table S1; HRTOFMS m/z 504.2877 [M+] (calcd for C_32_H_40_O_5_, 504.2870).

### Cell Viability

The effect of various compounds (**1–8**) on the viability of HT22 cells was determined by MTT assay. The cells were plated into 96-well plates at 105 cells per well containing 100 μl growth medium and were incubated for 24 h. Each compound with varying concentrations was added to each well. Further, MTT solution (5 mg/ml) was added to each well. The formazan precipitate formed was dissolved in 100 μl of dimethyl sulfoxide, and the cells were incubated for 2 h. Following incubation, the absorbance was measured using an automated microplate reader (Bio-Tek, USA) at 562 nm. The relative cell survival (%) was calculated as the ratio between absorbances of treated and control (untreated) cells, expressed as a percentage. The experiments were performed at least 3 times with each condition plated in triplicates.

### Microscopy

To observe morphological changes, glutamate (4 mM)-treated HT22 cells with or without MLG treatment were examined under a phase-contrast microscope (Olympus, Japan).

### Analysis of Apoptosis

The apoptotic cell population was evaluated by using an Annexin V APC/PI Apoptosis Detection Kit (BioLegend, USA). In brief, HT22 cells were seeded into a 6-well plate and treated with various concentrations of MLG. This was followed by exposure to 4 mM glutamate for 12 and 24 h. The cells were harvested, washed twice with PBS, and further stained with annexin V/PI for 30 min at 37°C and 5% CO_2_. Subsequently, the cells were analyzed using a flow cytometer (BD FACSVerse, BD Biosciences, USA). Data were analyzed using Flow Jo software.

### Mitochondrial Membrane Potential (Δψm)

MMP was measured using tetramethylrhodamine methyl ester perchlorate (TMRM) and MitoProbe JC-1 (5′,6,6′-tetrachloro-1,1′,3,3′-tetraethylbenzimidazolylcarbocyanine iodide; Thermo Fisher Scientific, USA) staining. The cells were seeded in a 6-well plate and treated with MLG followed by exposure to 4 mM glutamate for 12 and 24 h. After harvesting, cells were incubated with 100 nM cell-permeable fluorescent indicator TMRM for 20 min. To detect the Δψm with MitoProbe JC-1, the cells were incubated with 2 μM JC-1 at 37°C and 5% CO_2_ for 30 min. Samples stained with TMRM or JC-1 were washed with PBS and resuspended in PBS with 1% FBS. The samples were further analyzed on a flow cytometer (BD FACSVerse, BD Biosciences). The results were analyzed by FlowJo software (Becton, Dickinson and Company, USA).

### Intracellular ROS Assay

To measure the mitochondrial ROS levels in the cells, the MitoSOXTM Red Mitochondrial Superoxide Indicator Kit (Thermo Fisher Scientific) was used. In brief, MitoSOXTM Red reagent working stock (5 μM) was added to the cells which were pretreated with MLG and glutamate. Further, the cells were incubated for 10 min at 37°C in dark. The cells were washed and resuspended again in 500 μl PBS with 1% FBS. ROS levels were analyzed by measuring intracellular fluorescence using a flow cytometer (BD FACSVerse, BD Biosciences).

### Western Blot

For western blot, the cells were lysed with RIPA buffer containing 0.1 mg/ml phenylmethylsufony fluoride. The cell lysates were centrifuged at 13,000 ×*g* and 4°C for 10 min. The protein levels were quantified using the Bradford assay. The same amount of protein was subjected to 10% SDS-PAGE, transferred to a polyvinylidene difluoride membrane, and incubated with primary antibodies in TBST with 1% nonfat dry milk at 4°C. Specific primary antibodies against Bcl-2, pp38, p38, pJNK, JNK, IF-1, GAPDH, and α-tubulin were purchased from Santa Cruz Biotechnology (USA). Antibodies against cleaved PARP, AIF, HO-1, pERK, and ERK were purchased from Cell Signaling Technologies (USA). After washing with 1× TBST, membranes were incubated with anti-mouse or anti-rabbit horseradish-peroxidase-conjugated secondary antibody (Jackson Laboratory) for 1 h. The signals were detected by ImageQuant LAS mini (Fujifilm, Japan).

### Reverse Transcription Polymerase Chain Reaction (RT-qPCR)

HT22 cells were plated in 6-well plates and incubated with MLG in the presence of 4 mM glutamate. Total RNA was extracted with an RNeasy mini kit (Qiagen, Germany) as per the manufacturer’s protocol. cDNA was synthesized using the iScript cDNA Synthesis Kit (Bio-Rad, USA) with 1 μg total RNA and was amplified by PCR using specific primers. The PCR reaction mix contained 10 μl of iTaq Universal SYBR Green Supermix (Bio-Rad), 1 μl of PCR forward primer (10 μM), 1 μl of PCR reverse primer (10 μM), 1 μl of cDNA template, and 7 μl of ddH_2_O. The qRT-PCR conditions were as follows: 95°C for 10 min (denaturation) followed by 40 cycles of 95°C for 15 s and 60°C for 60 s. The procedures were performed using a StepOnePlus Real-Time PCR system (Thermo Fisher Scientific).

### Statistical Analysis

All values were expressed as means ± standard error. Statistical significance was determined by Student’s *t*-test. *p* < 0.05 was considered statistically significant.

## Results

### Structure Elucidation of a New Pterocarpan, 2-Geranylbicolosin A (1)

**Fig. 1:**
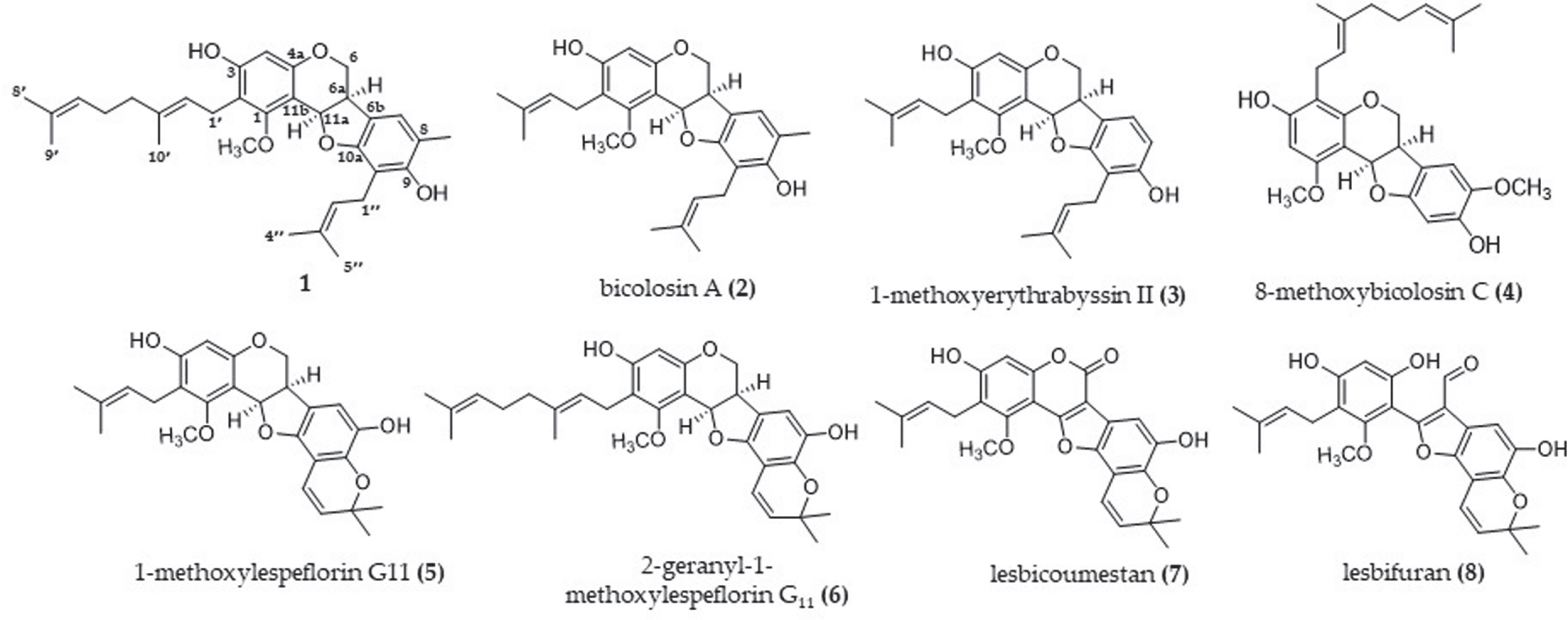
Structures of isoflavonoids from *Lespedeza bicolor* (1–8).

A new pterocarpan, 2-geranylbicolosin A (**1**), along with seven previously reported isoflavonoids, were isolated after a bioassay-guided fraction and chemical investigation of *L. bicolor*. Detailed comparison of the ^1^H and ^13^C NMR data of **1** with those of compound **2** (bicolosin A) indicated that 1 was a geranyl derivative of bicolosin A (Table S1). Placement of the geranyl side chain was confirmed at C-2 through HMBC correlations from H-1′ [δ_H_ 3.19 (2H, m)] to C-1 (δ_C_ 160.0), C-2 (δ_C_ 115.4), and C-3 (δ_C_ 157.9) and from H-2′ [δ_H_ 5.16 (1H, m)] to C-2 . A cis configuration of the protons H-6a and H-11a was observed on the basis of the ^1^H–^1^H coupling constant (*J* = 6.4 Hz) and ROESY experiment (Fig. S1). The absolute configuration of **1** was indicated as the R form, according to the positive Cotton effect (Δε +5.85) at 287 nm and negative optical rotation (${\left[\alpha \right]}_{D}^{20}$-163, c 0.21, MeOH), and compared with that of isolated pterocarpans, as reported earlier [15, 16]. Thus, compound **1** was named ‘2- geranylbicolosin A’ because it differed from bicolosin A only with regard to substitution of the geranyl group (Fig. 1).

### Neuroprotective Effect of Compounds Isolated from *L. bicolor* on Glutamate-Treated HT22 Cells

**Fig. 2:**
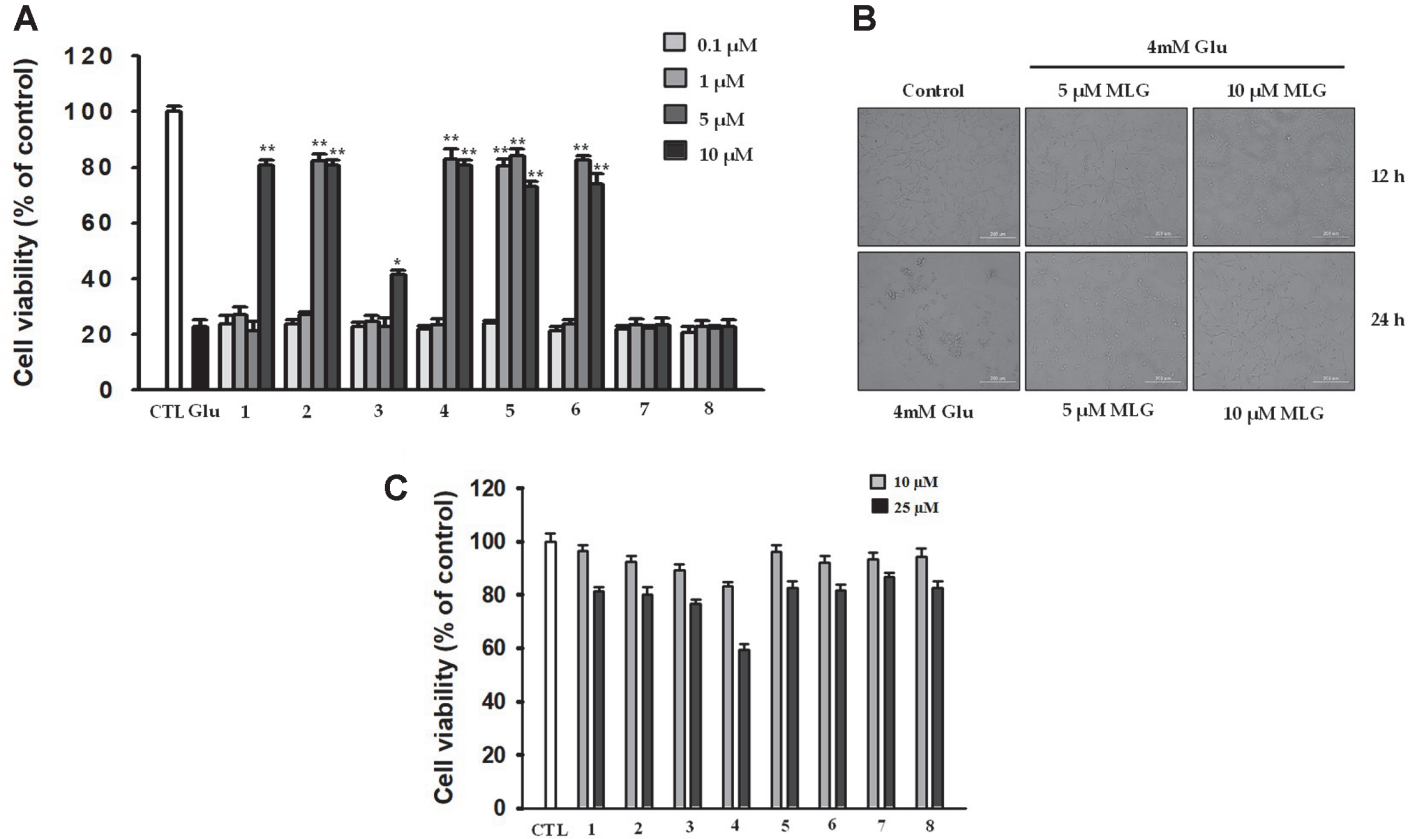
(**A**) Neuroprotective effects of compounds 1–8 in glutamate-treated HT22 cells. *p < 0.05, **p < 0.01 vs glutamate group. (**B**) The change in morphology was observed by microscopy (400× magnification). (**C**) Cytotoxicity of compounds 1–8 in HT22 cells.

To assess the neuroprotective effects of the isolated compounds, glutamate-treated HT22 cells were cultured with the isolated compounds (Fig. 1). Glutamate is a toxicant that induces oxidative stress in HT22 cells causing severe damage and neuroexcitotoxicity [17]. HT22 cells were exposed to glutamate (4 mM) in the presence of isolated compounds at the concentration ranging from 0.1 to 10 μM for 24 h (Fig. 2). The cell viability was determined using 3-(4,5-dimethylthizaol-2-yl)-2,5-diphenyltetrazolium bromide (MTT) assay and was expressed as the percentage of untreated control. After incubation with 4 mM glutamate for 24 h, the viability of HT22 cells decreased to 20.5% ± 0.1%, indicating that 4 mM glutamate caused significant toxicity. However, its toxic effects were diminished by several compounds obtained from *L. bicolor*; the structural requirements for the protective action were further analyzed. The arylbenzofuran-type compound (**8**) did not protect HT22 cells from glutamate-induced cell death; similarly, the coumestan-type compound (**7**) exhibited no protective effects at concentrations up to 10 μM. On the other hand, the pterocarpan-type compounds (**1–6**) exhibited significant neuroprotective effect against glutamate-neurotoxocity. Among them, 1-methoxylespeflorin G11 (MLG) (**5**), having a geranyl group at C-2, a prenyl group at C-10, and a methyl group at C-8, exhibited a strong protective effect with increasing cell viability (Fig. 2A). Additionally, microscopic analysis revealed that MLG strongly inhibited apoptosis induced by glutamate in HT22 cells (Fig. 2B). Besides, no significant cytotoxicity was observed at MLG concentrations up to 10 μM, whereas slight cytotoxicity was observed from 25 μM of MLG (Fig. 2C). Therefore, to further investigate the neuroprotective mechanism, we used MLG at 5 and 10 μM.

### Attenuation of Glutamate-Induced Apoptosis by MLG in HT22 Cells

**Fig. 3:**
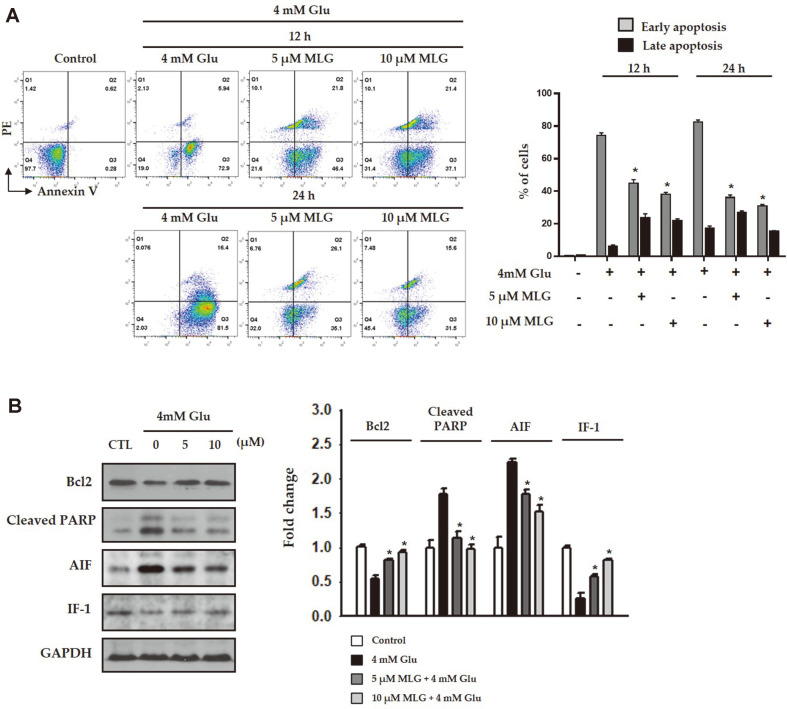
Inhibitory effect of MLG on glutamate-induced apoptosis. (**A**) The level of apoptosis was evaluated using annexin V/PI double staining. Cell death was assessed by flow cytometry. (**B**) Whole cell lysates from HT22 cells were immunoblotted with antibodies specific to Bcl-2, cleaved PARP, AIF, IF-1, and GAPDH. *p < 0.05 vs glutamate group.

To investigate the protective ability of MLG against glutamate-induced apoptosis, flow cytometric analysis was performed on the damaged cells probed with annexin V/propidium iodide (PI) (Fig. 3A). The proportions of early apoptotic cells were increased after exposure to glutamate for 24 h. However, when the cells were treated with 10 μM MLG prior to glutamate exposure, the proportions of early apoptotic cells were decreased as shown in Fig. 3A. These data demonstrated that MLG attenuated glutamate-mediated early apoptosis in HT22 cells as revealed by PI staining. In the western blot analysis, the level of the antiapoptotic protein Bcl-2 that was reduced after 4mM glutamate treatment was restored following 5 and 10 μM MLG treatment (Fig. 3B). On the other hand, the elevated level of AIF and cleavage of poly (ADP-ribose) polymerase (PARP), which promotes apoptosis by preventing DNA repair, were suppressed by MLG in a dose-dependent manner. IF-1 levels were not significantly different in cells treated with or without MLG.

### Prevention of Glutamate-Induced Intracellular ROS Generation and Mitochondrial Dysfunction by MLG

**Fig. 4:**
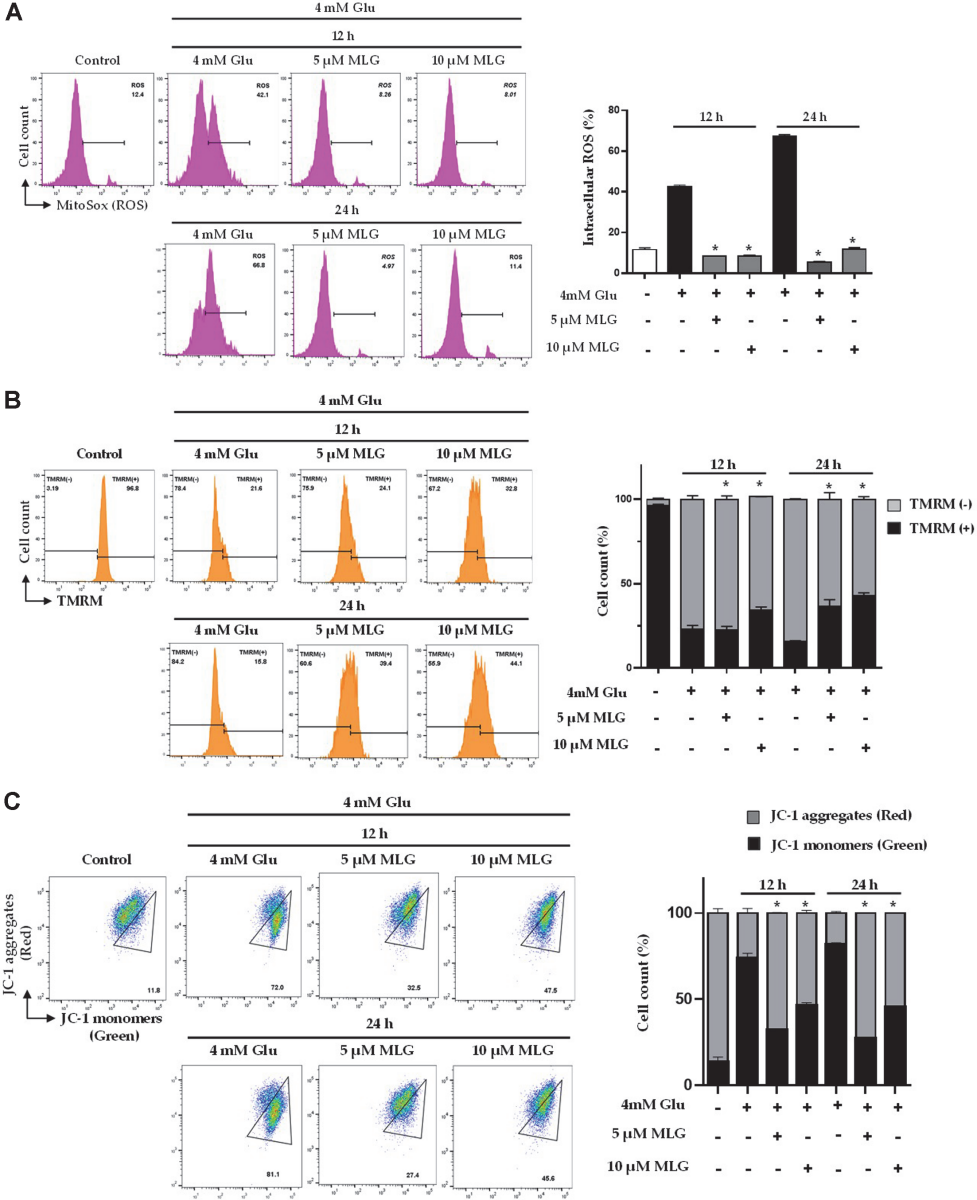
MLG attenuated glutamate-induced apoptosis via prevention of reactive oxygen species (ROS) generation and mitochondrial dysfunction. (**A**) ROS detection through measurement of fluorescence intensity using fluorescence microscopy. (**B**) MMP was analyzed using flow cytometry; mitochondria were stained with tetramethylrhodamine methyl ester perchlorate (TMRM) and (**C**) JC-1 staining. *p < 0.05 vs glutamate group.

Because oxidative stress is a major event during neuronal cell death, preventing ROS could be a strategy for the treatment of AD [18, 19]. We investigated mitochondrial ROS using flow cytometry through MitoSOXTM Red staining. Fig. 4A shows that the mitochondrial ROS level was significantly enhanced in the glutamate-treated HT22 cells than in the untreated control. The results demonstrated that MLG markedly inhibited the accumulation of intracellular ROS increased after glutamate treatment (Fig. 4A). Apoptosis-induced mitochondrial dysfunction was demonstrated by reduced mitochondrial membrane potential (MMP; ΔΨm). To determine the MMP, the cells were treated with MLG, stained with tetramethylrhodamine methyl ester perchlorate (TMRM), and analyzed using flow cytometry. Glutamate-treated cells demonstrated significant membrane potential depolarization after 24 h (Fig. 4B). The results showed that MLG prevented glutamate-induced disruption of MMP in a time- and dose-dependent manner. Moreover, the disruption of MMP was investigated using flow cytometry by JC-1 staining of cells treated with or without MLG under glutamate treatment (Fig. 4C). The glutamate-treated cells exhibited a more drastic decrease in MMP than the control group. JC-1 forms aggregates with red fluorescence in the mitochondria of normal cells and remains in the form of green fluorescent monomers in the cytosol of apoptotic cells, the latter indicating a decrease in MMP. As shown in Fig. 4C, MLG drastically improved the mitochondrial red/green fluorescence intensity ratio, indicating that MLG prevented glutamate-induced mitochondrial dysfunction.

### Suppression of Glutamate-Induced Apoptosis-Related Proteins and MAPK Activation by MLG

**Fig. 5:**
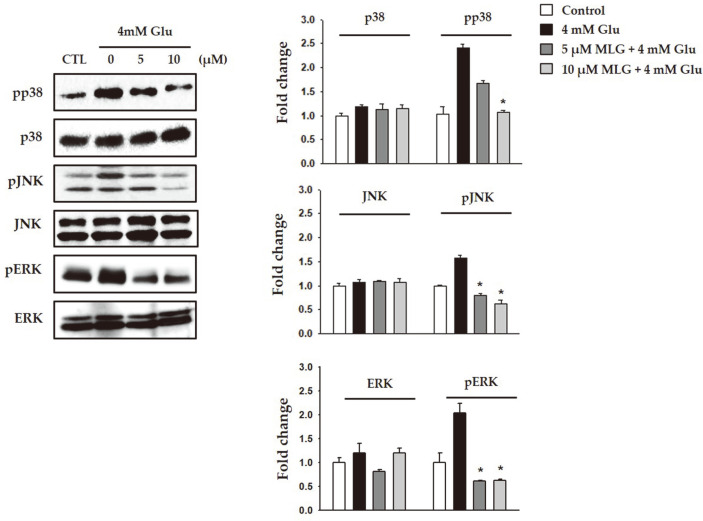
Effects of MLG on MAPK cascades in HT22 cells subjected to glutamate-induced cell death. Expression levels of p-JNK, JNK, p-ERK, ERK. *p < 0.05 vs glutamate group.

MAPK activation plays a critical role in the regulation of cell survival as well as death. Suppression of MAPK phosphorylation could be a protective mechanism against glutamate-induced neuronal cell death. Additionally, it has been reported that preventing intracellular ROS generation inhibited the phosphorylation of MAPKs and cell death, indicating the involvement of ROS-mediated phosphorylation of MAPKs in AD [19]. Therefore, we further evaluated the effects of MLG on the activation of MAPK through investigating the phosphorylated ERK, JNK, and p38 cascades. Treatment of HT22 cells with glutamate significantly induced the phosphorylated MAPK cascades. In contrast, MLG inhibited the phosphorylation of MAPKs induced by glutamate (Fig. 5). These results suggested that suppression of the phosphorylation of MAPKs was the underlying molecular mechanism of the MLG-mediated neuroprotective effect in glutamate-induced cell death in HT22 cells.

### Protection Against Glutamate-Induced Cytotoxicity in HT22 Cells by MLG Via HO-1 Induction

**Fig. 6:**
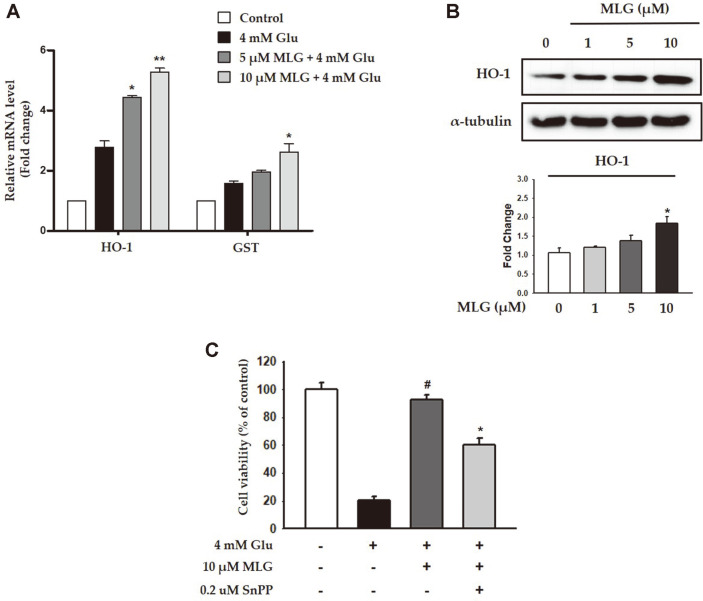
Effects of MLG on HO-1 expression in HT22 cells. (**A**) mRNA levels of HO-1 and GST were measured by RTqPCR. *p < 0.05 vs glutamate group. (**B**) Expression of HO-1 in presence of MLG at various concentrations was evaluated. *p < 0.05 vs glutamate group. (**C**) SnPP, an HO-1 inhibitor, inhibited the cytoprotective effects of MLG. HT22 cells were treated with 4mM glutamate and incubated with 0.2 μM SnPP or 10 μM MLG, followed by the measurement of HO-1 expression. #*p* < 0.05 vs glutamate group; *p < 0.05 vs glutamate- and MLG-treated group.

To investigate the antioxidant effects of MLG, we evaluated the mRNA levels of HO-1 and GST, which play a central role in the elimination of oxidized macromolecules. The mRNA level of HO-1 was noticeably increased in a dose-dependent manner, whereas the mRNA level of GST did not increase drastically (Fig. 6A). A previous study reported that HO-1 is attracting considerable attention for its potential neuroprotective effects in cell death models [20]. In this study, MLG treatment gradually increased the HO-1 expression in HT22 cells. We used SnPP, an HO-1 inhibitor, to investigate whether HO-1 is involved in glutamate-induced cytotoxicity in HT22 cells. As shown in shown in Figs. 6A and 6B, cotreatment with MLG and SnPP decreased the viability of HT22 cells as compared with treatment of MLG alone, indicating the involvement of HO-1 in MLG-mediated cytoprotective effect against glutamate-induced cytotoxicity.

## Discussion

In the present study, we investigated the neuroprotective effects of isoflavonoids obtained from *L. bicolor* on HT22 hippocampal cells. Among all the compounds, MLG, which has a pterocarpan moiety, effectively exhibited protective effects on HT22 cells even at low concentrations (Fig. 2). MLG significantly restored glutamate-induced morphological alteration and cell death as revealed in microscopic analysis (Fig. 2B). Glutamate increased intracellular ROS and mitochondrial dysfunction, which lead to AIF-dependent apoptosis in neuronal cells. As glutamate-induced HT22 cell death has the typical features of oxidative stress-induced programmed cell death, we investigated relevant cell death mechanisms in the time course experiments using Annexin V/PI staining. The result demonstrated that MLG markedly prevented glutamate-induced apoptotic cell death (Fig. 3A). The intrinsic apoptotic pathway is mitochondria-dependent, and it responds to various stress conditions such as excitotoxicity and oxidative stress. Therefore, mitochondria play an important role in the regulation of cell apoptosis. In line with morphological changes and apoptotic progress, low MMPs were observed in glutamate-treated HT22 cells. However, MLG prevented glutamate-induced disruption of MMP in a time- and dose-dependent manner (Fig. 4B). Furthermore, the expression levels of Bcl-2, PARP, AIF, IF, and MAPKs were determined by western blot analysis to study whether caspase modulation is involved in glutamate-induced apoptosis. MLG treatment resulted in an increase in the antiapoptotic protein Bcl-2 level and a decrease in cleaved PARP and AIF levels, which promote apoptosis (Fig. 3B). Additionally, the glutamate-induced phosphorylation of MAPKs, including pp38, pERK, and pJNK, was significantly reduced by pretreatment with MLG. Previous studies have reported that ROS generation could trigger cell apoptosis via initial mitochondrial membrane hyperpolarization that led to the collapse of the MMP [21]. To evaluate the effect of MLG on the glutamate-induced oxidative stress, intracellular ROS was measured using MitoSOXTM Red staining followed by flow cytometry (Fig. 4A). MLG markedly inhibited the accumulation of intracellular ROS increased by glutamate treatment; thus, MLG prevented intrinsic mitochondrial apoptosis in glutamate-treated HT22 cells by regulating the ROS- and caspase-dependent pathways. The mitochondrial apoptosis was assessed by JC-1 assay (Fig. 4C). JC-1 forms aggregates with red fluorescence in the mitochondria of normal cells and remains in the form of green fluorescent monomers in the cytosol of apoptotic cells, the latter indicating a decrease in MMP. As expected, MLG drastically recovered the mitochondrial red/green fluorescence intensity ratio.

HO-1 is a major antioxidant enzyme that plays an important role in protecting cells from oxidative stress via the reduction of ROS production. As the induction of HO-1 by several phytochemicals isolated from natural products has been widely recognized as an effective neuroprotective strategy, HO-1 expression by pharmacological modulators may represent a useful target for therapeutic intervention. In this study, MLG treatment gradually increased the HO-1 expression in HT22 cells. Additionally, cotreatment of MLG and SnPP decreased the viability of HT22 cells, indicating that the cytoprotective effects of MLG might be due to the induction of HO-1 expression.

Taken together, this study provided insights into the underlying mechanisms for MLG-induced neuroprotection in glutamate-induced apoptosis in HT22 cells. We demonstrated that MLG inhibited glutamate-induced apoptosis by eliminating ROS accumulation. Moreover, the cytoprotective effect of MLG in glutamate-treated cells was accompanied with HO-1 induction and reduction of MAPK activity. As ROS overproduction has gained increasing importance as a factor contributing to the development of neuronal diseases, MLG could serve as a promising natural agent for treating neurodegenerative diseases.

## Supplemental Materials



Supplementary data for this paper are available on-line only at http://jmb.or.kr.
