# Supplementary material for: 1-Methoxylespeflorin G11 Protects HT22 Cells from Glutamate-Induced Cell Death through Inhibition of ROS Production and Apoptosis
Source: J Microbiol Biotechnol. 2020 Dec 30;31(2):217–25. doi: 10.4014/jmb.2011.11032 (PMC9705990; doi:10.4014/jmb.2011.11032)
Supplement: Supplementary file 1 [file jmb-31-2-217-supple.pdf]

J. Microbiol. Biotechnol.
https://doi.org/10.4014/jmb.2011.11032

S1. Key COSY, HMBC, and ROESY correlations for structure determination of 2-geranylbicolosin A (**1**)

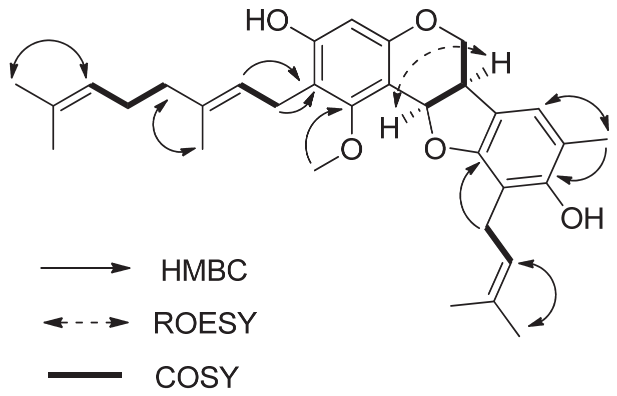

S2. ^{1}H NMR (400 MHz) spectrum of compound **1** in DMSO-d_6..

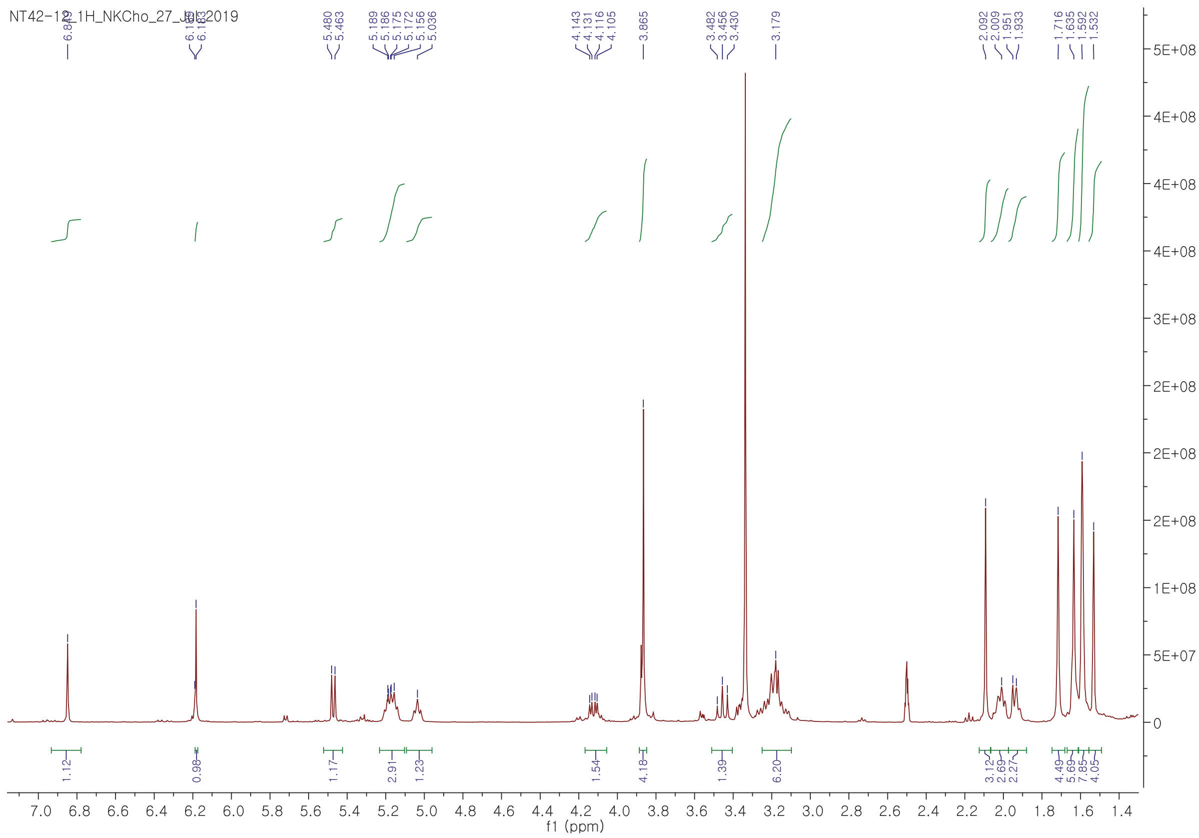

S3. ^{13}C NMR (100 MHz) spectrum of compound **1** in DMSO-d_6..

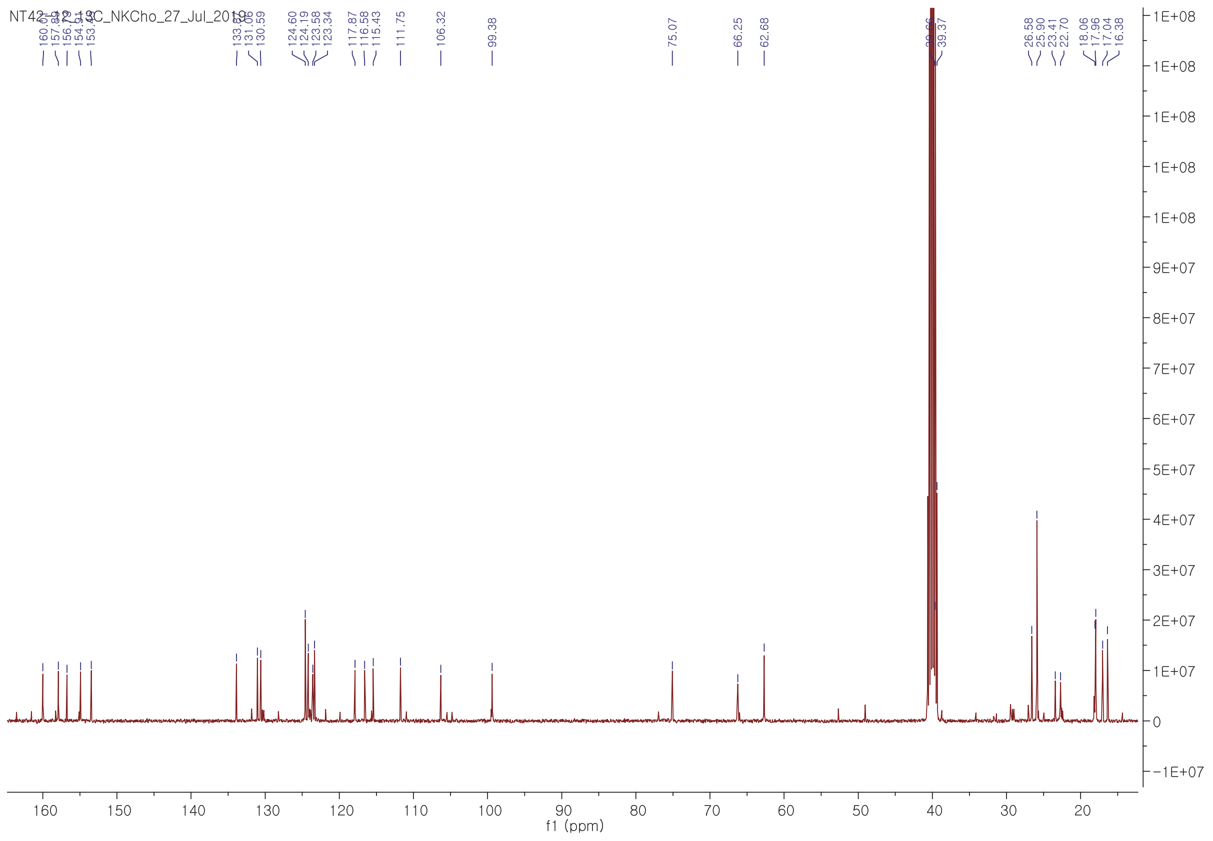

S4. ^{1}H-^{1}H COSY spectrum of compound **1** in DMSO-d_6.

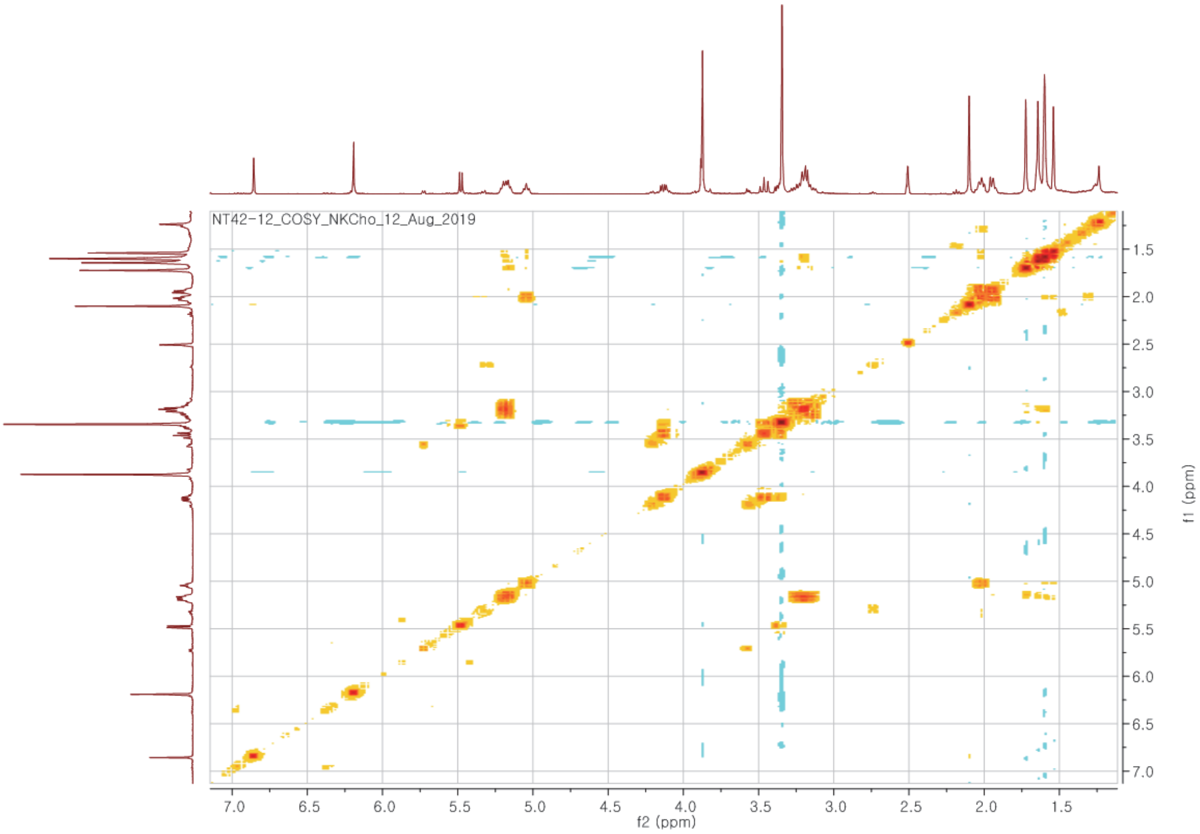

S5. HSQC spectrum of compound **1** in DMSO-d_6.

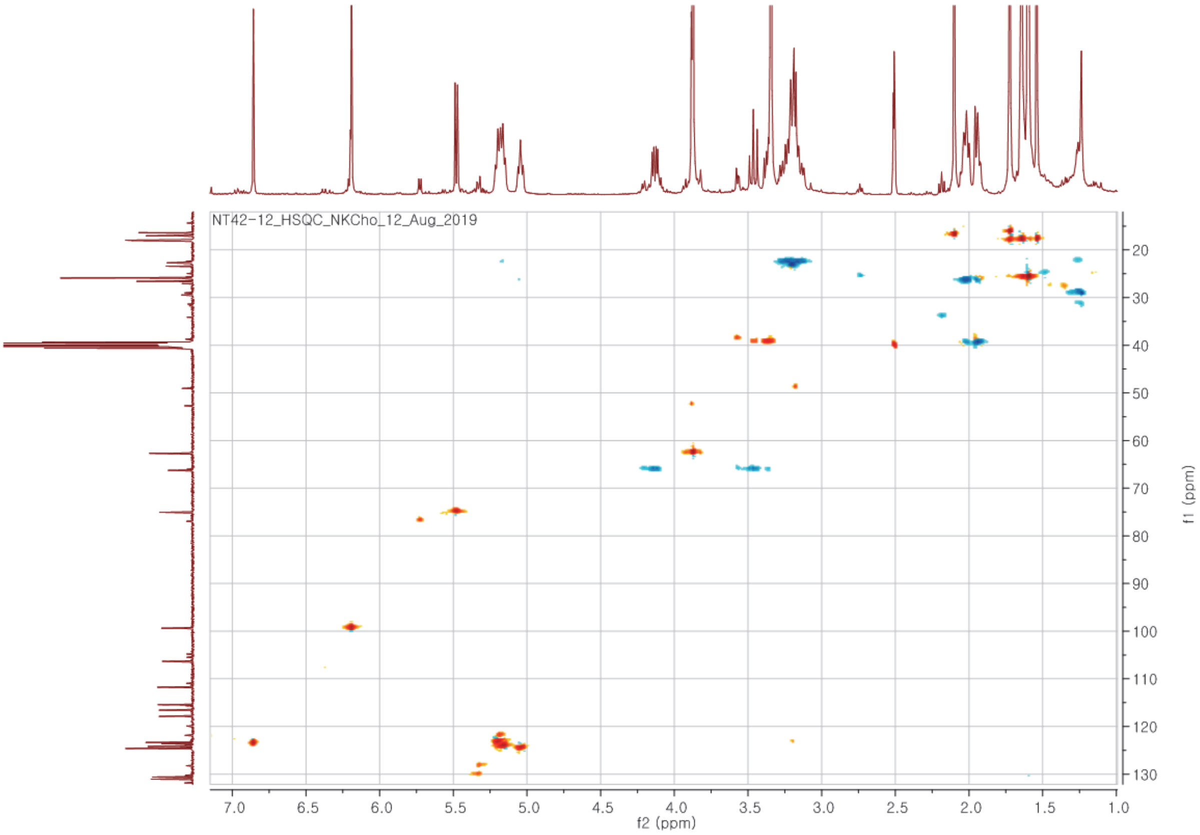

S6. HMBC spectrum of compound **1** in DMSO-d_6.

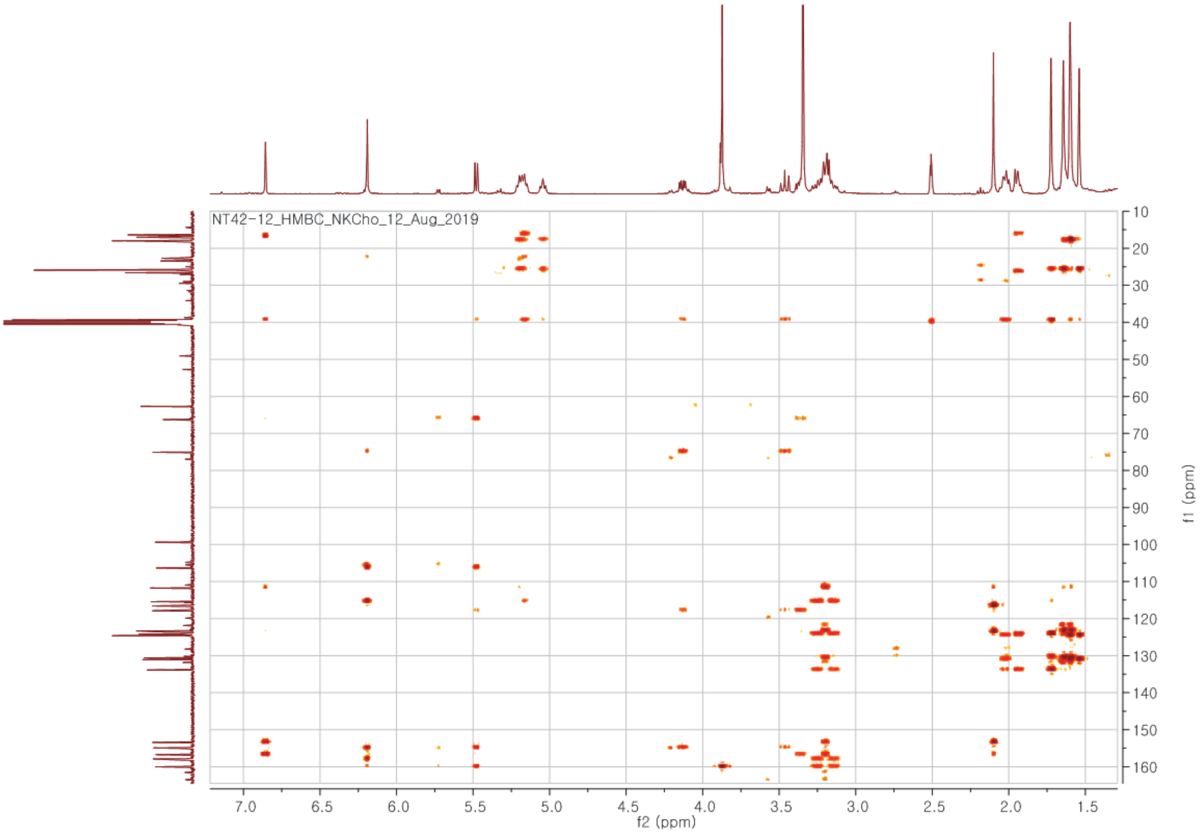

S7. ROESY spectrum of compound **1** in DMSO-d_6.

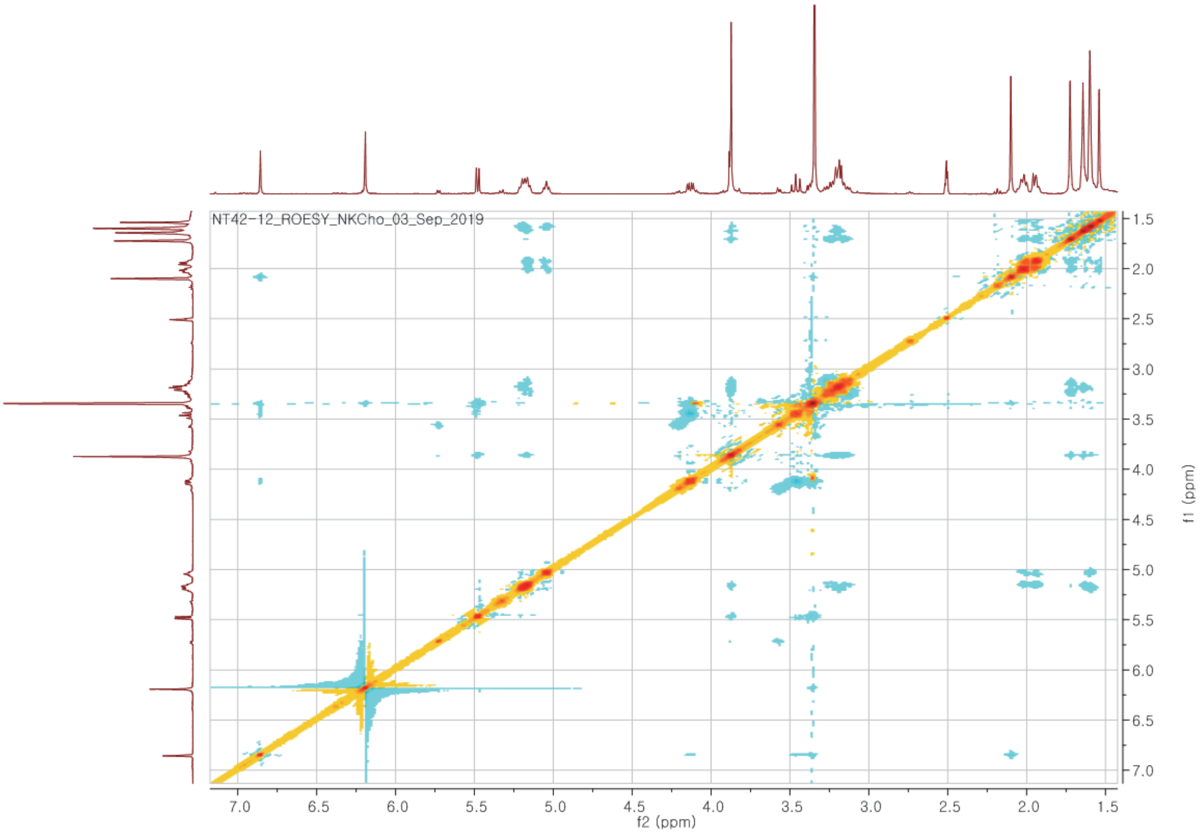

S8. HRTOFMS spectrum of compound **1** in DMSO-d_6.

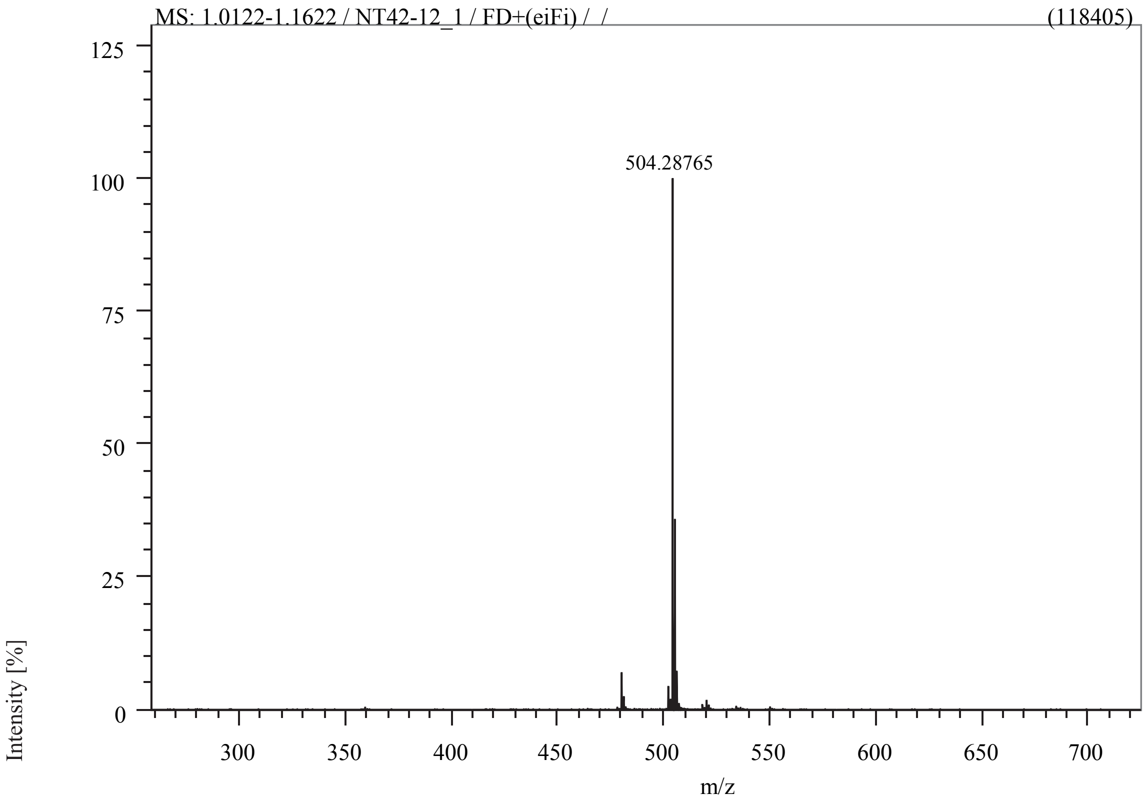

Elemental Composition

Parameters Elements Set 1:

		Symbol	C	H	O
Tolerance:	10.00 ppm	Min	0	0	0
Electron:	Odd/Even	Max	32	40	5
Charge:	+1				
DBE:	-90.0 - 90.0				

Results

Mass	Intensity	Formula	Calculated Mass	Mass Difference [mDa]	Mass Difference [ppm]	DBE
504.28765	118404.89	C32 H40 O5	504.28703	0.62	1.24	13.0

S9. CD spectrum of compound **1** in MeOH.

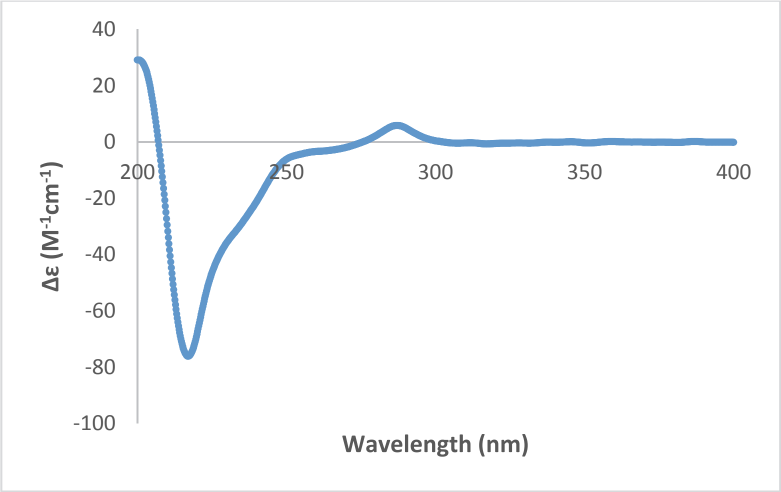

S10. Western blot analysis of cotreatment with MLG and SnPP in glutamate-treated HT22 cells

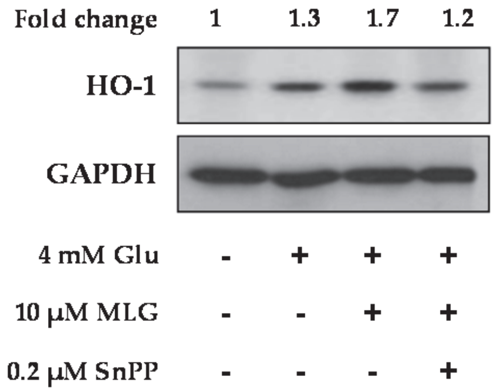

Table 1. ^1H NMR (400 MHz, DMSO-d_6) and ^{13}C NMR (100 MHz, DMSO-d_6) Spectroscopic Data for Compounds **1**.

**Position**	**1**	
	δ_C	δ_H (J in Hz)
1	160.0	
2	115.4	
3	157.9	
4	99.4	6.18, s
4a	154.9	
6	66.3	4.15, dd (4.4, 10.4); 3.46, t (10.4)
6a	39.4	3.36, m
6b	117.9	
7	123.6	6.85, s
8	116.6	
9	153.5	
10	111.8	
10a	156.7	
11a	75.1	5.52, d (6.4)
11b	106.3	
1′	22.7	3.19, m
2′	124.2	5.16, m
3′	133.9	
4′	39.7	1.93, m
5′	26.6	2.01, m
6′	124.6	5.04, m
7′	131.1	
8′	25.9	1.59, s
9′	18.0	1.53, s
10′	16.4	1.72, s
1″	23.4	3.18, m
2″	123.3	5.19, m
3″	130.6	
4″	25.9	1.59, s
5″	18.1	1.64, s
1-OCH_3	62.7	3.87, s
8-CH_3	17.0	2.09, s
